# Global Analysis of Predicted G Protein−Coupled Receptor Genes in the Filamentous Fungus, *Neurospora crassa*

**DOI:** 10.1534/g3.115.020974

**Published:** 2015-10-09

**Authors:** Ilva E. Cabrera, Itallia V. Pacentine, Andrew Lim, Nayeli Guerrero, Svetlana Krystofova, Liande Li, Alexander V. Michkov, Jacqueline A. Servin, Steven R. Ahrendt, Alexander J. Carrillo, Liza M. Davidson, Andrew H. Barsoum, Jackie Cao, Ronald Castillo, Wan-Ching Chen, Alex Dinkchian, Stephanie Kim, Sho M. Kitada, Taffani H. Lai, Ashley Mach, Cristin Malekyan, Toua R. Moua, Carlos Rojas Torres, Alaina Yamamoto, Katherine A. Borkovich

**Affiliations:** * Department of Plant Pathology and Microbiology, University of California, Riverside, California 92521; † Graduate Program in Genetics, Genomics and Bioinformatics, University of California, Riverside, California 92521; ‡ College of Natural and Agricultural Sciences, University of California, Riverside, California 92521

**Keywords:** filamentous fungi, G protein−coupled receptors, functional genomics, signal transduction, Heterotrimeric G proteins

## Abstract

G protein−coupled receptors (GPCRs) regulate facets of growth, development, and environmental sensing in eukaryotes, including filamentous fungi. The largest predicted GPCR class in these organisms is the Pth11-related, with members similar to a protein required for disease in the plant pathogen *Magnaporthe oryzae*. However, the Pth11-related class has not been functionally studied in any filamentous fungal species. Here, we analyze phenotypes in available mutants for 36 GPCR genes, including 20 Pth11-related, in the model filamentous fungus *Neurospora crassa*. We also investigate patterns of gene expression for all 43 predicted GPCR genes in available datasets. A total of 17 mutants (47%) possessed at least one growth or developmental phenotype. We identified 18 mutants (56%) with chemical sensitivity or nutritional phenotypes (11 uniquely), bringing the total number of mutants with at least one defect to 28 (78%), including 15 mutants (75%) in the Pth11-related class. Gene expression trends for GPCR genes correlated with the phenotypes observed for many mutants and also suggested overlapping functions for several groups of co-transcribed genes. Several members of the Pth11-related class have phenotypes and/or are differentially expressed on cellulose, suggesting a possible role for this gene family in plant cell wall sensing or utilization.

G protein−coupled receptors (GPCRs) are responsible for a diversity of cell functions, including environmental sensing, metabolism, immunity, growth, and development in eukaryotic cells (Bock *et al.* 2014; Chini *et al.* 2013). GPCRs are seven alpha-helical transmembrane (TM) proteins anchored in the plasma membrane, with an intracellular carboxyl- and extracellular amino-terminus (Shukla *et al.* 2014; Chini *et al.* 2013). The heterotrimeric G protein−signaling cascade initiates with the GPCR sensing the stimulus (ligand) and then transducing this signal to the inside of the cell via heterotrimeric G proteins (Tesmer 2010). Heterotrimeric G proteins are composed of three subunits (α, β, and γ) that are associated with the GPCR in the inactive state. Ligand binding to the GPCR causes a conformational change within the receptor that leads to exchange of GDP for GTP on the Gα subunit and dissociation of the Gα from the Gβγ heterodimer and the GPCR (Jastrzebska 2013; Tesmer 2010). Both the Gα-GTP and Gβγ can then regulate downstream effectors, which include mitogen-activated protein kinase cascades, ion channels, adenylyl cyclases, phosphodiesterases and phopholipases (Chini *et al.* 2013; Neves *et al.* 2002; Mende *et al.* 1998). The Gα subunit has native GTPase activity, hydrolyzing GTP to GDP. In addition, Regulators of G protein Signaling (RGS) proteins, also known as GTPase-Activating Proteins, can accelerate the hydrolysis process by 2000-fold (Ross and Wilkie 2000). Subsequently, the Gα-GDP and Gβγ reassociate to form the inactive heterotrimeric complex at the GPCR on the membrane.

*Neurospora crassa* has served as a eukaryotic model for human and plant pathogenic fungi and has been the most-studied filamentous fungal species for the past hundred years [reviewed in (Davis and Perkins 2002; Borkovich *et al.* 2004; Selker 2011)]. *N. crassa* has a more complex life cycle than budding or fission yeasts and grows through extension, branching, and fusion of tube-like structures called hyphae [reviewed in (Springer 1993; Borkovich *et al.* 2004; Glass *et al.* 2004)]. *N. crassa* hyphae contain incomplete cross-walls (septa) that delineate cell compartments but still allow flow of organelles and cytoplasmic materials throughout the intertwined hyphal structure (mycelium). *N. crassa* possesses two different asexual sporulation pathways, with one (macroconidiation) under the control of a circadian rhythm, blue light, reactive oxygen species (ROS) and other environmental stimuli (Springer 1993; Greenwald *et al.* 2010; Baker *et al.* 2012). *N. crassa* also has a well-studied sexual cycle, involving two mating types, male and female partners, and production of numerous cell types, culminating in elaboration of the fruiting body that contains the meiotic progeny (Bistis 1981; Raju 1992; Nelson 1996; Kim *et al.* 2012). *N. crassa* offers several advantages for genetic analysis, including more than 1000 mapped morphologic mutations and a nearly complete gene knockout collection (Dunlap *et al.* 2007; Borkovich *et al.* 2004; Perkins *et al.* 2001).

Heterotrimeric G proteins and GPCRs have been demonstrated to regulate growth and development in *N. crassa* (Baasiri *et al.* 1997; Ivey *et al.* 1996; Kays and Borkovich 2004; Kays *et al.* 2000; Kim and Borkovich 2004; Kim *et al.* 2012; Krystofova and Borkovich 2005, 2006; Li and Borkovich 2006; Won *et al.* 2012; Li *et al.* 2007). *N. crassa* possesses three Gα, one Gβ, and one Gγ subunit(s), and 10 putative GPCRs were originally annotated in the *N. crassa* genome sequence (Borkovich *et al.* 2004; Galagan *et al.* 2003). Later annotation of GPCR genes in the filamentous fungal pathogen *Magnaporthe oryzae* demonstrated evidence for three additional GPCR classes in filamentous fungi (Kulkarni *et al.* 2005).

Predicted *N. crassa* GPCRs fall into 12 of the 13 classes (I-XII) described for *Verticillium* and *Trichoderma* fungal species (Zheng *et al.* 2010; Gruber *et al.* 2013) (Table 1), with a fourteenth class [Pth11-related; (Kulkarni *et al.* 2005); see Neurospora crassa *GPCR families shared with other eukaryotes*] named Class XIV for this study. To date, only five of the 43 predicted *N. crassa* putative GPCR genes have been functionally characterized. Classes I and II contain the pheromone response receptors PRE-2 and PRE-1 (Kim and Borkovich 2004; Kim *et al.* 2012). These two GPCRs have been demonstrated to interact with peptide pheromones to regulate female fertility in a mating-type dependent manner (Kim and Borkovich 2004, 2006; Kim *et al.* 2002, 2012). The *N. crassa* Class III GPCR is GPR-4, which regulates growth on poor carbon sources (Li and Borkovich 2006). Class V is the cAMP receptor-like (CRL) group containing GPR-1, GPR-2, and GPR-3 (Borkovich *et al.* 2004; Krystofova and Borkovich 2006), which are similar to chemoattractant GPCRs found in *Dictyostelium discoideum* (Klein *et al.* 1988). There is no evidence that fungal CRLs bind cAMP. *N. crassa* GPR-1 is localized in female reproductive structures and is required for normal formation of perithecial beaks and spore discharge from female fruiting bodies (Krystofova and Borkovich 2006). Class IX includes the microbial opsins NOP-1 and ORP-1. Heterologous experiments showed that NOP-1 is a light-activated retinal-binding protein (Bieszke *et al.* 1999b). Loss of *nop-1* leads to subtle alterations in conidiation and expression of conidiation-specific genes in *N. crassa* (Bieszke *et al.* 2007; Bieszke *et al.* 1999a). However, functions for ORP-1 have not been described.

**Table 1:** *Neurospora crassa* G protein−coupled receptor gene families and summary of growth/developmental and chemical sensitivity/nutrition phenotypes

Fungal GPCR Class	Description GPCR Class	NCU*^a^*	*N. crassa* Gene(s)*^b^*	*Saccharomyces cerevisiae* Homolog*^c^*	Aspergillus nidulans Homolog*^d^*	Linear Growth*^e^*	Asexual Development*^f^*	Sexual Development*^g^*	Chemical Sensitivity*^h^*/ Nutrition*^i^*
I	Fungal Pheromone	05758	*pre-2*	*STE2*	*gprA*		AH	PP, P, A*^j^*	
II	Fungal Pheromone	00138	*pre-1*	*STE3*	*gprB*			P, A*^j^*	
III	Carbon Sensory	06312	*gpr-4*	*GPR1*	None		AH		
IV	Stm1-like	00300	*gpr-5*	*YPQ1*	AN5720				C
IV	Stm1-like	09195	*gpr-6*	*RTC2*	AN5720				C
V	cAMP Receptor-Like	00786	*gpr-1*	None	AN8262		AH	P	C
V	cAMP Receptor-like	04626	*gpr-2*	None	AN8262		AH	P	
V	cAMP Receptor-like	09427	*gpr-3*	None	AN8262		AH	P	
VI	GprK-like/RGS Domain	09883	*gpr-7*	None	AN7795 (*gprK*)				F
VII	Rat growth hormone releasing factor receptor-like	03253	*gpr-8*	None	AN6680	NA*^k^*			
VIII	mPR-Like/PAQR	03238	*gpr-9*	*IZH3*	AN4932				S
VIII	mPR-like/PAQR	04987	*gpr-10*	*IZH2*	AN4932				F
IX	Microbial Opsin	10055	*nop-1*	*YRO2*, *MRH1*, *HSP30*	AN3361		AH		M
IX	Microbial Opsin	01735	*orp-1*	*YRO2*, *MRH1*, *HSP30*	AN3361		AH		
X	Lung 7TM Superfamily	00182	*gpr-11*	*PTM1*	AN0063				YE
XI	GPCR89/ABA GPCR	00005	*gpr-12*	YHR078w	None				
XII	Family C-like	06629	*gpr-13*	None	AN8601				
XIII	DUF300 superfamily/Ps GPR11	06987	*gpr-14*	YKR051W	AN2232	NA*^k^*			
XIV*^l^*	Pth11-like	00700	*gpr-15*	None	AN12202	R		PP, P	
	Pth11-like	02903	*gpr-16*	None	AN0178		AH		F
	Pth11-like	04106	*gpr-17*	None	AN7774		AH		
	Pth11-like	04931	*gpr-18*	None	AN10886	NA*^k^*			
	Pth11-like	05101	*gpr-19*	None	AN1930	NA*^k^*			
	Pth11-like	05187	*gpr-20*	None	AN8661	NA*^k^*			
	Pth11-like	05189	*gpr-21*	None	AN8661				
	Pth11-like	05307	*gpr-22*	None	AN0751		AH		
	Pth11-like	05829	*gpr-23*	None	AN0751	R	AH		C,T
	Pth11-like	05854	*gpr-24*	None	AN8971				FL
	Pth11-like	06531	*gpr-25*	None	AN5639				C
	Pth11-like	17171	*gpr-26*	None	AN5664	NA*^k^*			
	Pth11-like	07538	*gpr-27*	None	AN5664				F
	Pth11-like	16721	*gpr-28*	None	AN2249	NA*^k^*			
	Pth11-like	07649	*gpr-29*	None	AN8943	R	AH		S,C
	Pth11-like	07769	*gpr-30*	None	AN4452		AH		
	Pth11-like	08429	*gpr-31*	None	None	I	AH		F
	Pth11-like	08431	*gpr-32*	None	AN5664				A
	Pth11-like	08447	*gpr-33*	None	AN5664				
	Pth11-like	08624	*gpr-34*	None	AN0178				
	Pth11-like	08718	*gpr-35*	None	AN8951				
	Pth11-Like	09022	*gpr-36*	None	AN5664				A
	Pth11-like	09201	*gpr-37*	None	AN5664				
	Pth11-like	09796	*gpr-38*	None	AN8328	R			
	Pth11-like	09823	*gpr-39*	None	AN1930		AH		A

GPCR, G protein−coupled receptor; NCU, *N. crassa* gene number.

a Based on the Broad Institute’s *Neurospora crassa* database (http://www.broadinstitue.org/annotation/genome/neurospora/MultiHome.html).

b *N. crassa* gene names are from the literature or designated during this study.

c Yeast orthologs were obtained after a Blastp search of the NCBI database, using a cut-off score of e-05 or less.

d *A. nidulans* orthologs were obtained from a Blastp search of the NCBI database, using a cut-off score of e-05 or less. Only the top hit is shown.

e R, reduced growth rate; I, increased growth rate relative to wild type.

f Defects in aerial hyphae (AH) or conidial development (C).

g Defects in protoperithecial (PP), perithecial (P), and/or ascospore (A) development.

h Chemical sensitivity phenotypes are indicated based on sensitivity or resistance to sorbitol (S), cytochalasin A (C), benomyl (B), tert-butyl hydroperoxide (T), menadione (M), FK506 (F), and fludioxonil (FL).

i Represented by increased or decreased growth on Avicel (A) or Yeast Extract (Y) compared with wild type.

j Mating type dependent.

k Not available; see Table S1 for details.

l Category XIV named in this study.

A recent study reported functional information for 15 GPCRs corresponding to Classes I-IX in the filamentous fungus *Aspergillus flavus* (Affeldt *et al.* 2014). However, as mentioned previously, 38 of the 43 identified putative GPCRs have not been functionally characterized in *N. crassa* (Table 1). Of these, Class IV contains GPR-5 and GPR-6, similar to Stm1 from the yeast *Saccharomyces cerevisiae* (Borkovich *et al.* 2004; Chung *et al.* 2001). Likewise, classes VI (RGS domain; one gene), VII (similar to rat growth hormone releasing factor receptors; 7tm_1 domain; one gene), VIII (similar to human steroid receptor; HLyIII domain; one gene), X (Lung 7_TM superfamily; one gene), XI (GPCR89/ABA GPCR; one gene), XII (Family C-like; one gene), XIII (DUF300 superfamily; one gene), and XIV (Pth11-related; 25 genes) were previously annotated but have not been functionally studied in *N. crassa* (Gruber *et al.* 2013; Li *et al.* 2007; Lafon *et al.* 2006; Kulkarni *et al.* 2005).

In this work, we systematically analyze available *N. crassa* knockout mutants lacking annotated GPCR genes to determine their growth, developmental, and chemical sensitivity phenotypes. We take advantage of existing messenger RNA (mRNA) profiling datasets to mine expression trends for several GPCR genes during growth and development. We demonstrate phenotypes for many of the uncharacterized mutants, as well as novel chemical phenotypes for some previously studied mutants. Importantly, our study is the first to probe functions for the large family of Pth11-related GPCRs in fungi.

## Materials and Methods

### Media and strains

Vogel’s minimal medium [VM; (Vogel 1964)] was used to support vegetative growth and development. Sexual differentiation was promoted by culturing on synthetic crossing medium (SCM) plates (Westergaard and Mitchell 1947). Colony formation was facilitated by growth on sorbose-containing medium plates (Davis and Deserres 1970). Where indicated, hygromycin B (Calbiochem, San Diego, CA) was used at a concentration of 200 µg/mL in media. Media were inoculated using conidia propagated on VM agar slants.

From the Fungal Genetics Stock Center (FGSC; Kansas City, MO), we obtained wild-type strains ORS-SL6a (FGSC 4200; *mat a*) and 74-OR23-IVA (FGSC 2489; *mat A*). Available GPCR homokaryotic mutants were obtained from the FGSC or created in our laboratory (see Supporting Information, Table S1 for strain information). A total of seven mutants were not available at the time of this study (Table S1). All *N. crassa* gene numbers are preceded by the prefix “NCU”. Knockouts of NCU03253 (*gpr-8*), NCU05101 (*gpr-19*), and NCU05187 (*gpr-20*) were attempted, but failed, either due to an inability to obtain knockout constructs or *N. crassa* primary transformants. The gene structures for NCU17171 (*gpr-26*) and NCU16721 (*gpr-28*) were reannotated after mutant construction, and the available knockout mutants (former NCU06891 and NCU07591, respectively) are incorrect.

Knockout mutants that were deposited at the FGSC as heterokaryons (NCU09796, NCU00700, and NCU08429) were purified to homokaryons using a genetic cross to wild-type strain FGSC2489, as described (Colot *et al.* 2006). All putative homokaryons were checked for the presence of the knockout cassette using gene-specific and *hph* primers in diagnostic polymerase chain reaction as described (Ghosh *et al.* 2014).

### Growth, developmental, chemical sensitivity, and nutritional phenotypes

Phenotypic assays were conducted essentially as previously described (Ghosh *et al.* 2014; Park *et al.* 2011b; Turner 2011), except that race tubes were prepared with 25-mL plastic disposable pipets (White and Woodward 1995). Some phenotypic tests used in earlier studies were omitted, including assessment of pigmentation and aerial hyphae formation on yeast extract-containing medium. For quantitative growth and developmental assays (apical extension rate and aerial hyphae height), mutants that exhibited a ±5 mm/d difference relative to wild type were considered significant (Colot *et al.* 2006; Park *et al.* 2011b). For detailed analysis of beak formation in the CRL mutant class of GPCR mutants, strains were inoculated on SCM (Westergaard and Mitchell 1947) plates and incubated under constant light for 1 wk at 25°. Cultures were fertilized using conidia from a wild-type strain of opposite mating type at 7 d postinoculation. Formation of perithecia was scored 1 wk after fertilization and beak morphology and ascospore ejection 2 wk after fertilization. Images of perithecia were captured using a SZX9 stereomicroscope (Olympus) and a digital camera. Delayed ascospore ejection and beak formation was checked after an additional week. Wild-type strain (FGSC 4200; *mat a*) was used as a control.

For chemical sensitivity screens, mutants were inoculated at the edge of 60 × 15-mm VM plates with and without chemical. The following chemicals and concentrations were assayed: sodium chloride (0.35 M), sorbitol (0.8 M), cytochalasin A (40 ng/mL; Sigma, St. Louis, MO), benomyl (92 ng/mL; Fluka, St. Louis, MO), tert-butyl hydroperoxide (0.13 mM; Sigma), Menadione (100 mM; Sigma), FK-506 (50 ng/mL; LC Laboratories, Woburn, MA), and fludioxonil (2.75 ng/mL; a gift from Frank Wong and Allison Tally). Sorbitol and sodium chloride were added to VM agar medium prior to autoclaving, while all other chemicals were added to cooled autoclaved VM agar medium using filter-sterilized concentrated stock solutions. Plates were incubated in the dark for 20−22 hr at 30°. VM plates lacking chemical were used as controls. The percent growth on the chemical was determined by dividing the colony radius for the plate containing the chemical by that of a VM plate. Four biological replicates were used in each of three independent experiments. Student’s *t*-test (paired, two-tailed) was used to identify mutants with significantly different sensitivity relative to wild type. A mutant was considered different from wild type if *P* ≤ 0.05 in at least two experiments and ≤0.20 in all three experiments. Mutants with better growth on a chemical or supplement/carbon source relative to wild type were scored as resistant or increased (R or I; Table 2), while slower-growing mutants were scored as sensitive or decreased (S or D; Table 2).

**Table 2:** G protein−coupled receptor mutants with chemical sensitivity and nutritional phenotypes

NCU	*N. crassa* Gene	Sorbitol	Peroxide	Menadione	FK506	Cytochalasin A	Fludioxonil	Avicel	Yeast Extract
00300	*gpr-5*					R (+29%)			
09195	*gpr-6*					R (+25%)			
00786	*gpr-1*					S (-13%)			
09883	*gpr-7*				R (+31%)				
03238	*gpr-9*	R (+11%)							
04987	*gpr-10*				R (+15%)				
10055	*nop-1*			R (+26%)					
00182	*gpr-11*								I (+18%)
02903	*gpr-16*				R (+18%)				
05829	*gpr-23*		S (-25%)			S (-29%)			
05854	*gpr-24*						S (-29%)		
06531	*gpr-25*					R (+25%)			
07538	*gpr-27*				R (+19%)				
07649	*gpr-29*	R (+21%)				S (-28%)			
08429	*gpr-31*				R (+16%)				
08431	*gpr-32*							I (+39%)	
09022	*gpr-36*							I (+47%)	
09823	*gpr-39*							I (+30%)	

Mutants were considered significantly different than wild type if the results of a T-test yielded *P* ≤ 0.20 for all three replicates and *P* ≤ 0.05 for at least two of the three replicates. For chemical sensitivity assays, R = more resistant than wild type and S = more sensitive than wild type. For nutritional phenotypes (yeast extract and avicel), I = increased growth relative to wild type. The percent difference relative to wild type is shown in parentheses for affected mutants. NCU, *N. crassa* gene number.

The GPCR mutants also were tested for nutritional phenotypes using 60 × 15-mm VM agar plates. Avicel (crystalline cellulose; Sigma) was substituted for sucrose in VM medium at a concentration of 2%. Yeast extract was added to VM medium to a concentration of 2% w/v. Incubation and measurement of plates and identification of mutants that were significantly different than wild type was determined as described for chemical sensitivity screening, above. Mutants with better growth on Avicel or 2% yeast extract relative to wild type were scored as increased (I; Table 2), whereas slower-growing mutants were scored as decreased (D; Table 2).

### Clustering of *N. crassa* GPCR expression data and heatmap generation

Expression data were mined for GPCR genes from four different datasets. Each dataset pertained to a specialized tissue type during development or an environmental condition. RNA-sequencing data were obtained for sexual development (Wang *et al.* 2014), whereas microarray data were utilized for colony development (Kasuga and Glass 2008) and a time course of conidiation (Greenwald *et al.* 2010). Lastly, RNA sequencing data for expression using 4 hr liquid cultures grown on either sucrose or the alternative carbon source Avicel (Coradetti *et al.* 2012) were downloaded from (http://www.ncbi.nlm.nih.gov/geo; Accession number GSE35227). To visualize the expression data, heatmaps were generated with the pheatmap package (V1.0.2); (Kolde 2015) using R (v3.1.1); (R Core Development Team 2014). Expression data for each gene was standardized using the included scaling function in pheatmap.

### Phylogenetic analysis of putative Pth11-related proteins

The Pth11 protein sequence from *Magnaporthe oryzae* and the 25 Pth11-related *N. crassa* GPCR protein sequences were aligned with T-coffee using default parameters and trimmed using TrimAl with a threshold of 0.7 (Notredame *et al.* 2000; Capella-Gutierrez *et al.* 2009). The programs Seqboot, Protpars, and Consense, belonging to the Phylip package were used to generate a consensus parsimony tree using 100 bootstrap replicates (Felsenstein 1989).

### Data availability

Strains are available from the FGSC or our laboratory upon request. File S1 contains detailed descriptions of phenotypic data.

## Results

### *Neurospora crassa* GPCR families shared with other eukaryotes

Mammalian GPCRs have been classified using the A-F classification scheme (Attwood and Findlay 1994) and more recently, the GRAFS method (Fredriksson *et al.* 2003; Schioth and Fredriksson 2005). The A-F classification contains six major superfamilies of GPCRs: Class A (rhodopsin like), Class B (secretin like), Class C (metabotropic glutamate/pheromone), Class D (fungal pheromone), Class E (cAMP receptors), and Class F (Frizzled/Smoothened family). The GRAFS classification grouped the human GPCRs into five families: Glutamate (Class C), Rhodopsin (Class A), Adhesion (Class B), Frizzled/Taste2 (Class F), and Secretin (Class B). A recent analysis provided evidence for four of the five GRAFS families (secretin is absent) in fungi (Krishnan *et al.* 2012). This work also provided evidence that the fungal cAMP and rhodopsin families share a common ancestor. Other studies have proposed up to 14 classes of GPCRs in fungi (Kulkarni *et al.* 2005; Zheng *et al.* 2010; Gruber *et al.* 2013).

Previous studies have identified 43 putative GPCR genes in the *N. crassa* genome (Li *et al.* 2007; Borkovich *et al.* 2004; Galagan *et al.* 2003) (Table 1). Of the GPCR families found in mammals, *N. crassa* possesses one Adhesion family GPCR (GPR-1; (Krystofova and Borkovich 2006) and two cAMP family GPCRs (GPR-2, GPR-3; (Borkovich *et al.* 2004; Galagan *et al.* 2003). *N. crassa* also possesses a mPR class (Class VIII), with homology to seven-helix progestin receptors in mammals (Thomas *et al.* 2007). These proteins are members of the progesterone and AdipoO receptor (PAQR) group of proteins found in many eukaryotes. Evidence that these proteins can function as bona-fide GPCRs in filamentous fungi is suggested by studies in *Sporothrix schenckii*, which show that progesterone is a ligand for a PAQR that also interacts with a Gα protein (Gonzalez-Velazquez *et al.* 2012). The *N. crassa* genome also predicts one seven-helix protein containing a Lung_7-TM_R domain (Liu *et al.* 2004) with homology to *S. cerevisiae* PTM1, a protein of unknown function (Inadome *et al.* 2005). Finally, *N. crassa* possesses one protein with weak similarity to Family C-like (metabotropic glutamate/pheromone) GPCRs in chicken (Zheng *et al.* 2010).

We and others have previously identified proteins similar to predicted *N. crassa* GPCRs in the yeast *S. cerevisiae* and the filamentous fungus *Aspergillus nidulans* (Lafon *et al.* 2006; Li *et al.* 2007) (Table 1). The results show that *S. cerevisiae* possesses proteins similar to 12 of the *N. crassa* predicted GPCRs, with members in classes I-IV, VIII-XI, and XIII, and an additional member (total of three proteins) in the microbial opsin class (Table 1). *A. nidulans* and *N. crassa* share homologs for most predicted *N. crassa* genes, with the exception of the Class XI GPCR89/ABA related gene *gpr-12* and one Pth11-related GPCR, *gpr-31* (Table 1).

Pth11 is a seven-helix transmembrane protein required for development of the infectious appressorium structure and pathogenesis in *M. oryzae* (DeZwaan *et al.* 1999). It has recently been demonstrated that Pth11, the Gα protein MagA, the RGS Rgs1, and the adenylyl cyclase Mac1 are co-localized on late endosomes during the early stages of pathogenesis, which would enable effective signal transmission (Ramanujam *et al.* 2013). Sequencing of numerous fungal genomes revealed that filamentous fungi contain a large number of proteins with homology to Pth11 (Kulkarni *et al.* 2005; Li *et al.* 2007; Zheng *et al.* 2010; Gruber *et al.* 2013), including 25 in *N. crassa* (Kulkarni *et al.* 2005; Li *et al.* 2007). This was particularly surprising in the case of *N. crassa*, since the wide array of genome defense mechanisms in this species is thought to have limited the number of gene families (Galagan *et al.* 2003). The presence of a large number of Pth11-related proteins suggests that members of this class serve important functions in *N. crassa*.

### Mutants with phenotypes during asexual growth and development

We had previously produced knockout mutants for 10 predicted GPCR genes in our laboratory [(Kim and Borkovich 2004; Kim *et al.* 2012; Bieszke *et al.* 1999a; Krystofova and Borkovich 2006; Li and Borkovich 2006); S. Krystofova, M. Nemcovic, L. Li, A. Michkov and K. Borkovich, unpublished data.]. Mutation of all predicted GPCR genes was attempted by the Neurospora Genome Project (Colot *et al.* 2006; Dunlap *et al.* 2007; Park *et al.* 2011a). Mutants for seven genes were not available (see the section *Materials and Methods*), leaving us with 36 viable mutants to analyze.

**Figure 1:**
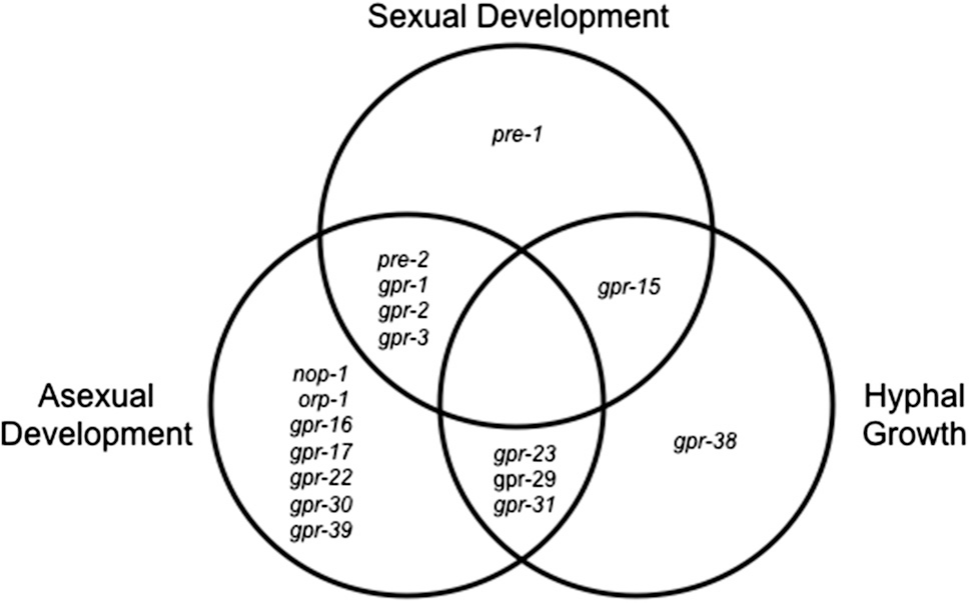
Venn diagram displaying G protein−coupled receptor mutants with phenotypes in growth and development. From the 36 available mutants, 17 (47%) exhibited a defect in at least one assay for hyphal growth or asexual or sexual development, as shown. Mutants with defects are indicated as gene names for the deleted genes.

We analyzed the 36 available GPCR knockout mutants for a variety of growth and developmental phenotypes (Table 1; Figure 1; Table S1), first focusing on hyphal growth and asexual sporulation (macroconidiation). The macroconidiation (conidiation) pathway is induced when *N. crassa* is exposed to oxygen (plate cultures) or to a variety of environmental stresses during growth in submerged culture [reviewed in (Springer 1993; Davis and Perkins 2002; Borkovich *et al.* 2004)]. The pathway begins with formation of aerial hyphae, tube-like structures that rise up roughly perpendicular to the substratum. Aerial hyphae form constrictions between cell compartments at their tips that eventually lead to formation of mature conidia that can be dispersed by mechanical perturbation or wind currents (Springer 1993).

We obtained quantitative data for hyphal growth rate and aerial hyphae height and qualitative data for conidia production (Table 1; Table S1). We identified a total of five knockout mutants (14%) with defects in extension of basal hyphae (Figure 1; Table 1; Table S1). Of interest, all five belong to Class XIV (Pth11-like). Δ*gpr-31* was the only mutant among the group that had an increased growth rate relative to wild type; all others (Δ*gpr-15*, Δ*gpr-23*, Δ*gpr-29*, and Δ*gpr-38*) grew more slowly than wild type.

A total of 14 GPCR mutants (39%) had defects in asexual development, specifically in formation of aerial hyphae (Figure 1; Table 1; Table S1). There were no GPCR mutants with obvious defects in conidia production. Of the mutants with aerial hyphae defects, 57% (8/14) are characterized as Class XIV (Pth11-like). Among the 14 mutants with aerial hyphae defects, half had a reduced phenotype, whereas the other half an increased aerial hyphae phenotype. Finally, of the five mutants with defects in basal hyphal growth, three also exhibited reduced formation of aerial hyphae (Δ*gpr-23*, Δ*gpr-29*, and Δ*gpr-31*).

### Mutants with defects in sexual development

Low nitrogen, as found in SCM, induces development of female reproductive structures (protoperithecia) (Westergaard and Mitchell 1947; Raju 1992) approximately 7 d postinoculation. Fertilization occurs when a specialized hyphae (trichogyne) from the protoperithecium fuses with a cell from an opposite mating type cell (male). After nuclear fusion, meiosis is followed by a round of mitosis and sexual spores (ascospores) are then formed within the mature perithecium.

This study analyzed three phases in the sexual cycle: protoperithecia, perithecia, and ascospore formation. A total of six GPCR mutants (17%) showed a defect in sexual development (Figure 1, Table 1; Table S1). As previously reported by our group (Kim and Borkovich 2004, 2006; Kim *et al.* 2012), the two pheromone receptor mutants displayed defects in sexual development, with *pre-2* affecting all three phases (protoperithecia, perithecia, and ascospore formation), and *pre-1* affecting perithecia and ascospore formation. However, it is should be noted that the sexual defects of these mutants are mating-type dependent; Δ*pre-2* is only female-sterile in *mat a*, and Δ*pre-1* in *mat A* (Kim and Borkovich 2004; Kim *et al.* 2012) (Table 1). The other four GPCR mutants with sexual defects (Δ*gpr-1*, Δ*gpr-2*, Δ*gpr-3*, and Δ*gpr-15*) all exhibited phenotypes in the perithecial phase, with Δ*gpr-15* having both a protoperithecial and perithecial defect. Despite their perithecial phenotypes, these four mutants still produced ascospores and thus are technically female-fertile (Table 1; Table S1). Thus, allowing for the mating type specificity of the pheromone receptor mutations, the results showed that no single GPCR gene was essential for female fertility in *N. crassa*.

### Mutants with defects in multiple phases of the lifecycle

The aforementioned results revealed that 17 of the 36 predicted GPCR mutants (47%) exhibited at least one growth or developmental phenotype (Table 1). There were no GPCR mutants with defects in all three phenotypic categories, but eight mutants possessed phenotypes in two categories (Figure 1; Table 1). The four mutants with defects in both asexual and sexual development included the Δ*pre-2* pheromone receptor mutant and those lacking any one of the three CRL receptor genes (*gpr-1*, *gpr-2*, and *gpr-3*). The remaining four mutants with defects in two categories (hyphal growth and either asexual or sexual development) were all in the Pth11-related Group (Figure 1; Table 1). The relatively low number of GPCR mutants with defects in more than one of the analyzed stages suggests either specialization, participation in pathways not uncovered during our study, or gene redundancy in the predicted GPCRs.

### The CRL class of predicted GPCRs is required for normal formation of perithecial beaks and ascospore ejection

**Figure 2:**
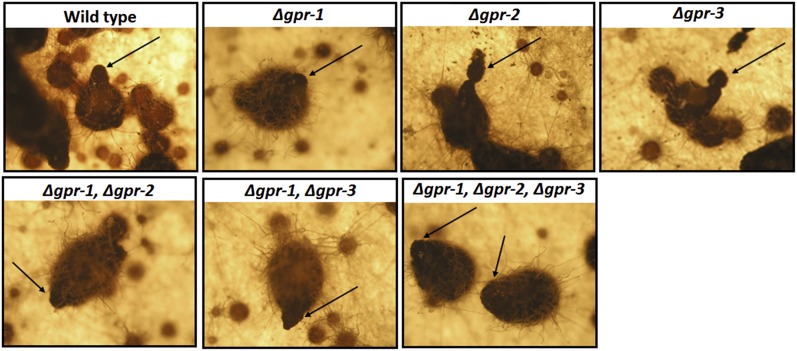
Perithecial development in Δ*gpr-1*, Δ*gpr-2*, and Δ*gpr-3* mutants lacking CRL class G protein−coupled receptorr. Synthetic crossing medium cultures of the indicated strains containing protoperithecia were fertilized using opposite mating type conidia from a wild-type strain. Perithecia were photographed 10 d later. The arrows indicate normal perithecial beaks (wild type), perithecia with beaks that lack ostioles (Δ*gpr-1*) or perithecia with beaks that bend downward or are torn during ascospore ejection (single, double or triple mutants lacking *gpr-2* or *gpr-3*).

We were interested to find that all three CRL (Class V) mutants possessed defects in both asexual and sexual development (Table 1). Notably, *gpr-1* was reported previously to have an important role in proper formation of perithecial beaks (Krystofova and Borkovich 2006). Beaks are structures formed at the tip of perithecia that are positively phototrophic to blue light and which normally contain an opening (ostiole) from which the mature ascospores are ejected (Harding and Melles 1983; Harris *et al.* 1975). Δ*gpr-1* beaks are deformed and lack ostioles, leading to rupture of the perithecium during discharge of ascospores (Krystofova and Borkovich 2006). Based on the phenotype of Δ*gpr-1* mutants, we decided to explore beak formation in Δ*gpr-2* and Δ*gpr-3* single mutants, as well as mutants lacking *gpr-1* and various combinations of the other two CRL genes (Figure 2).

As shown previously, Δ*gpr-1* beaks do not form ostioles and therefore cannot eject ascospores (Figure 2; data not shown). Loss of *gpr-2* or *gpr-3* leads to formation of beaks that bend downward and/or are torn during ascospore ejection (Figure 2). Similarly, mutants lacking *gpr-1* in combination with *gpr-2* and/or *gpr-3* form beaks that are not positively phototrophic and do not contain ostioles (Figure 2). Thus, all three CRL genes influence beak morphology and ascospore discharge in *N. crassa*.

### Chemical sensitivity and nutritional screens reveal additional GPCR mutants with phenotypes

As shown in our previous studies with *N. crassa* protein kinase and phosphatase mutants (Park *et al.* 2011b; Ghosh *et al.* 2014) and in work with the yeast *S. cerevisiae* (Hillenmeyer *et al.* 2008), chemical sensitivity and nutritional screening is a useful tool to uncover functions for mutants that lack observable growth or developmental defects. With this in mind, we conducted chemical/nutritional screens on the 36 GPCR mutants, using a total of 10 chemicals. Mutants were grown on agar medium with and without the added chemical, the percent growth determined and compared to wild type (see the section *Materials and Methods*). Mutants with relative growth rates statistically greater than or less than wild type were categorized as resistant or sensitive, respectively (Table 2). The chemicals used induced osmotic stress [sorbitol and NaCl; (Ivey *et al.* 1996)], oxidative stress [menadione and tert-butyl hydrogen peroxide; (Belden *et al.* 2007; Kato *et al.* 2004)], cytoskeletal disruption [cytochalasin A and benomyl; (Cooper 1987; Willhite 1983)], inhibition of the Ca^2+^-calmodulin dependent phosphatase calcineurin [FK506; (Prokisch *et al.* 1997)], fungicidal activity [fludioxonil; (Ochiai *et al.* 2001)], and nutritional substitution or supplementation (Avicel and yeast extract).

No mutants were significantly different than wild type during growth on NaCl or benomyl, and therefore these two agents are not shown in Table 2. A total of 18 GPCR mutants exhibited at least one phenotype using the other eight chemicals in the screen and 11 of these mutants did not exhibit a growth or developmental phenotype. More than half (10/18; 56%) of the 18 mutants with chemical phenotypes are classified as Pth11-like receptors.

The chemical that yielded the largest number of mutants with phenotypes was cytochalasin A, with six affected strains (Table 2). Cytochalasin A interferes with actin function by inhibiting polymerization of monomers (Cooper 1987). Three mutants (Δ*gpr-5*, Δ*gpr-6*, and Δ*gpr-25*) were found to be resistant to cytochalasin A, while Δ*gpr-1*, Δ*gpr-23*, and Δ*gpr-29* strains were sensitive. GPR-5 and GPR-6 are similar to Stm1p, a PQ-loop family protein originally implicated in nitrogen sensing upstream of the Gα protein Gpa2 in *S. pombe* (Chung *et al.* 2001), with more recent evidence suggesting that the *S. cerevisiae* homolog YPQ1 may function as a vacuolar amino acid transporter (Sekito *et al.* 2014). The finding that two Stm1p-related proteins confer resistance to cytochalasin A in *N. crassa* suggests a connection between actin polymerization and G protein signaling or vacuolar amino acid transport. GPR-1 is a CRL, whereas *gpr-23*, *gpr-25*, and *gpr-29* encode Pth11-related proteins. Of these, GPR-25 is the closest *N. crassa* homolog to *M. oryzae* Pth11 (Figure 6). The cytochalasin A phenotype for Δ*gpr-25* mutants may point to a role for GPR-25 in actin dynamics.

FK506 inhibits the phosphatase calcineurin, which is regulated by Ca^2+^-calmodulin. Five GPCR mutants, Δ*gpr-7*, Δ*gpr-10*, Δ*gpr-16*, Δ*gpr-27*, and Δ*gpr-31*, were all resistant to FK506, suggesting a possible role in regulation of the phosphatase or modulating calcium signaling. Of interest, the closest yeast homolog for *N. crassa* GPR-10 is Izh2p (Table 1). Izh2p and three other proteins in yeast each have four metal ion binding sites and have been variously implicated in zinc homeostasis, regulation of membrane sterol content and a signaling pathway upstream of the Zap1 transcription factor (Lyons *et al.* 2004). Izh2p is of particular interest, since it has recently been shown to function as a receptor for plant PR-5 proteins and is required for the response to polyene antifungal drugs (Narasimhan *et al.* 2005; Villa *et al.* 2011). Our finding that the five *N. crassa* mutants, including Δ*gpr-10*, are resistant to FK506 suggests that the calcineurin phosphatase may be a component of the their downstream pathways.

Two GPCR mutants, *Δgpr-9* and *Δgpr-29*, showed increased resistance to osmotic stress induced by sorbitol. GPR-9 is most homologous to the *S. cerevisiae* protein Izh3p, a mPR-like PAQR class protein similar to Izh2p, whereas GPR-29 is a Pth11-related receptor. Izh3p dosage affects resistance to polyene drugs in yeast, similar to Izh2p (Villa *et al.* 2011).

Peroxide, menadione, and fludioxonil testing each yielded one GPCR with a phenotype (Δ*gpr-23*, Δ*nop-1*, and Δ*gpr-24*). We observed that Δ*nop-1* mutants are resistant to the ROS generating compound menadione. The only phenotypes that had previously been noted for Δ*nop-1* mutants were effects on colony morphology (Bieszke *et al.* 1999a) and conidiation-regulated gene expression (Bieszke *et al.* 2007). The menadione phenotype is of particular interest since a link between conidiation and ROS has been determined for *N. crassa*, with elevated ROS correlating with aerial hyphae and conidia development (Hansberg *et al.* 1993). Our demonstration that Δ*nop-1* mutants are more resistant to menadione correlates with the slightly increased formation of aerial hyphae in these mutants in plate cultures and with elevated expression of certain conidiation specific genes at specific times during asexual development (Bieszke *et al.* 2007).

We also explored growth of the mutants in medium supplemented with yeast extract or with crystalline cellulose (Avicel) as an alternative carbon source. Yeast extract is rich in vitamins, amino acids and peptides and we have previously used this supplement to recover knockout mutants with defects in amino acid biosynthetic pathways (Colot *et al.* 2006). Δ*gpr-11* mutants grew better than wild type in medium containing yeast extract (Table 2). This was the only phenotype noted for this Lung7_TM superfamily protein during our study. Three Pth11 class mutants, Δ*gpr-32*, Δ*gpr-36*, and Δ*gpr*-39, grew better than wild type on medium containing 2% Avicel as an alternative to sucrose (30–47% greater; Table 2). This finding suggests that these Pth11 class proteins may participate in sensing plant-related carbohydrates.

### Predicted GPCR genes are differentially expressed during growth and development

**Figure 3:**
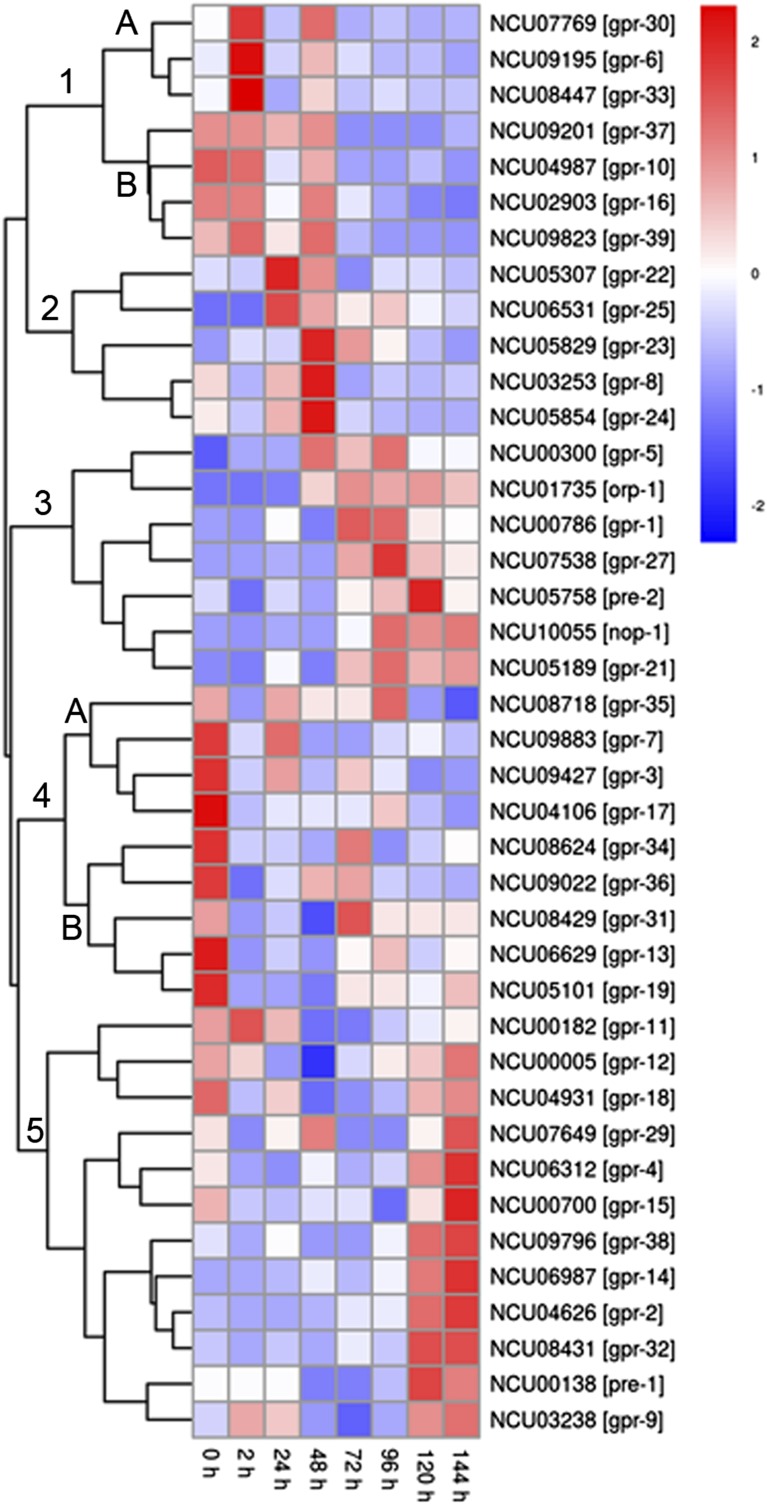
Clustering and heatmap generation of mRNA expression data for *N. crassa* G protein−coupled receptor (GPCR) genes during a time course of sexual development. RNAseq data were mined from (Wang *et al.* 2014). Expression data for 37 of the 43 predicted GPCR genes was contained in the dataset and a heat map prepared as described in the section *Materials and Methods*. Red color denotes greater levels of expression, whereas blue corresponds to lower expression.

**Figure 4:**
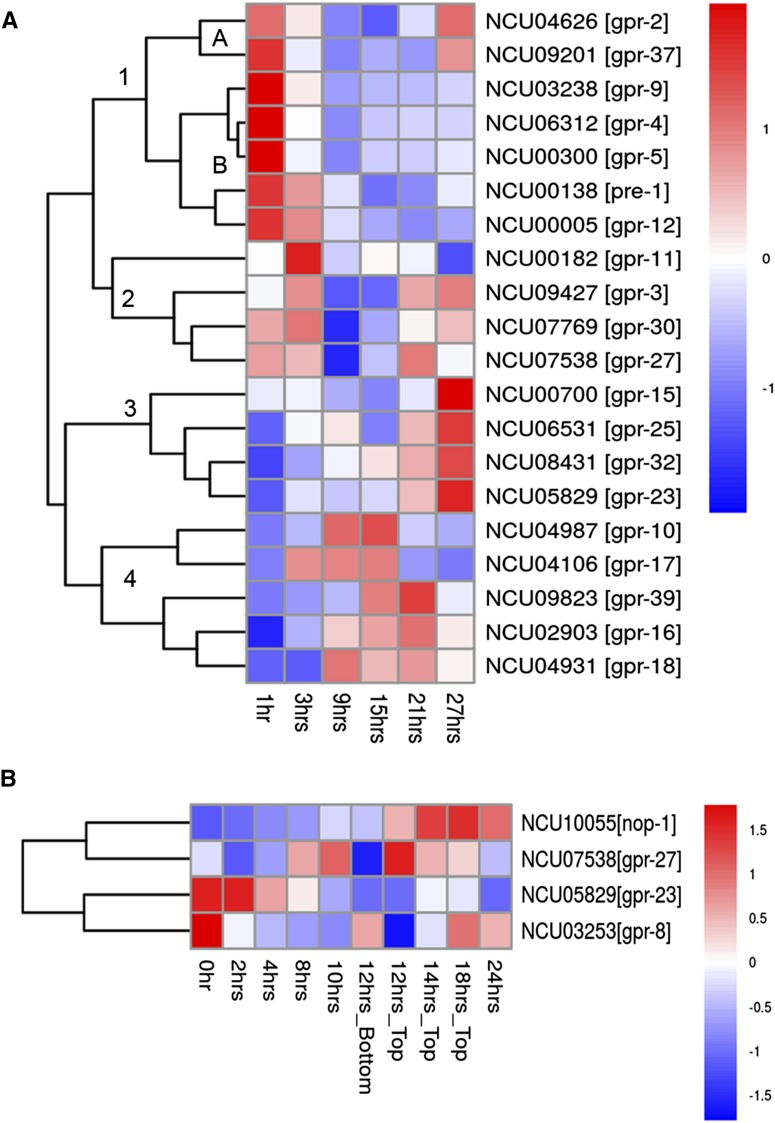
Clustering and heatmap generation of mRNA expression data for *N. crassa* G protein−coupled receptor (GPCR) genes during time courses of colony growth and asexual development (conidiation). Data were taken from the indicated sources and heat maps generated as described in the section *Materials and Methods*. Red color denotes greater levels of expression, whereas blue corresponds to lower expression. (A) Gene expression during colony growth. Microarray data were obtained from (Kasuga and Glass 2008) for 20 predicted GPCR genes expressed during colony growth. (B) Gene expression during conidiation. Microarray data were mined from (Greenwald *et al.* 2010) for four predicted GPCR genes expressed during asexual development (conidiation).

**Figure 5:**
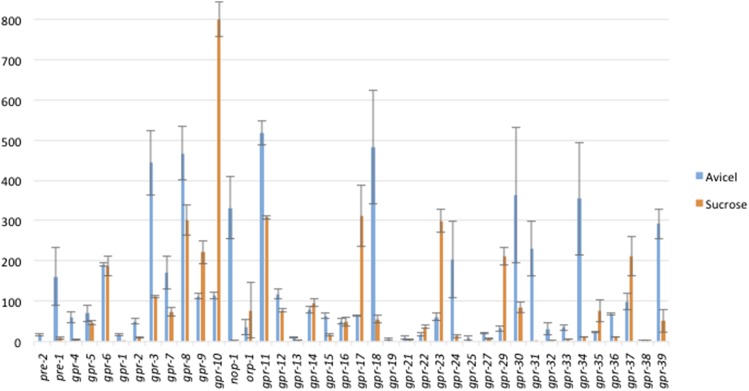
Expression patterns for 40 predicted G protein−coupled receptor genes on two different carbon sources. RNAseq data were obtained from Coradetti *et al.* (2012) for genes expressed in tissue grown for 4 hr in liquid medium containing either sucrose (orange bars) or Avicel (crystalline cellulose; blue bars) as a carbon source. Y-axis is reads per kilobase per million mapped reads, whereas x-axis indicates genes.

RNA expression data for GPCRs was mined from publically available datasets. Experiments included those performed under different environmental conditions and time-courses for specialized tissue types (Kasuga and Glass 2008; Greenwald *et al.* 2010; Wang *et al.* 2014; Coradetti *et al.* 2012; Nordberg *et al.* 2014). In all instances, data were analyzed and visualized using pheatmap (V1.0.2) (Kolde 2015) (Figure 3, Figure 4, and Figure 5).

We first analyzed an RNAseq dataset for sexually differentiating cultures that included eight time-points (0, 2, 24, 48, 72, 98, 120, and 144 hr after fertilization) (Wang *et al.* 2014). At time 0 hr, cultures contain vegetative hyphae and unfertilized female structures (protoperithecia). Within 24 hr after fertilization, perithecia can be observed. Croziers, structures that will develop into asci containing the meiotic progeny (ascospores) become apparent between 48 and 72 hr after fertilization (Raju 1992; Read and Beckett 1996). After 96 hr, asci become detectable and at 120−144 hr perithecia produce beaks, a structure required to eject ascospores into the environment.

The dataset for sexual development contained expression data for 37 of the 43 predicted GPCR genes, with the genes falling into five major groups (Figure 3). Group 1 genes are all down-regulated after 72 hr, at the time when the synchronization of nuclei is occurring in the croziers. Group 1 can be subdivided into subgroups 1A and 1B, which differ in their patterns of expression before 72 hr. The three genes in Subgroup 1A (*gpr-6* and two Pth11-related genes) are up-regulated at 2 hr after fertilization, with expression diminishing at 24 hr, rising again at 48 hr. Cluster 1B genes (*gpr-10* and three Pth11-related GPCRs) are highly expressed at 0 and 2 hr after fertilization, but then resemble Subgroup 1A for the rest of the time course.

Group 2 genes (*gpr-8* and four Pth11-related GPCRs) exhibited the greatest levels of expression at 24 or 48 hr after fertilization (the time when perithecia first become visible), with relatively lower levels at 72−144 hr. Group 3 genes (seven total) have relatively low levels of expression until 48−72 hr, corresponding to the emergence of croziers. In general, Group 3 gene expression remains high throughout the rest of perithecial development. Three Group 3 genes have been previously examined for expression during at least one phase of sexual development using northern analysis: *pre-2*, *gpr-1*, and *nop-1*. In congruence with the RNAseq data, expression of the pheromone receptor *pre-2* is low in *mat A* protoperithecia (0 hr), but increases after fertilization with *mat a* conidia (Kim *et al.* 2012). Northern analysis demonstrated that The CRL GPCR *gpr-1* is highly expressed in protoperithecia, with even greater levels in perithecia (Krystofova and Borkovich 2006) and the RNAseq results showed that mRNA levels are greater during the time of perithecial development than in protoperithecia (Figure 3). Finally, although the microbial opsin *nop-1* is highly expressed in protoperithecia, levels in perithecia have not been investigated using northern analysis (Bieszke *et al.* 1999a), precluding direct comparison to the RNAseq results.

Group 4 (nine genes) is divided into Subgroups 4A and 4B (Figure 3). In general, genes in both subgroups exhibit their highest levels of expression at 0 hr, prior to fertilization. Group 4A genes have dropped to their lowest levels of expression by 120−144 hr, whereas expression of most Group 4B genes rises again later during perithecial development. The authors of the RNAseq study noted that due to the difficulty in obtaining pure reproductive structures, vegetative hyphae is likely present in the samples before 48 hr postfertilization (Wang *et al.* 2014). With this in mind, it is important to note that mutants lacking three of the genes in Group 4 had a phenotype during growth or sexual or asexual development. Δ*gpr-3* has defects in asexual and sexual development; Δ*gpr-31* in asexual development and linear growth; and Δ*gpr-17* only in asexual development (Table 1, Figure 1).

Group 5 is the largest, with 12 genes (Figure 3). Most of the genes in this group are up-regulated 120−144 hr after fertilization, when mature perithecia are present. However, only the CRL *gpr-2* and the pheromone receptor *pre-1* play obvious roles during sexual development (Table 1). The finding that the other 10 genes have no obvious sexual cycle phenotype suggests at least some of them may be functionally redundant. Finally, the expression trend for *pre-1* is supported by previous northern analysis revealing increased expression of *pre-1* in *mat A* protoperithecia after fertilization with *mat a* conidia (Kim and Borkovich 2004).

We next mined microarray expression data from six time points during vegetative colony development (1, 3, 9, 15, 21, and 27 hr) (Kasuga and Glass 2008). Colony establishment consists of multiple events: hyphal extension, branching, anastomosis, and asexual sporulation (Kasuga and Glass 2008). Early features of colony establishment can be seen in the 1-hr time point at the leading hyphal edge. This “periphery growth zone” is rich with organelles, such as endoplasmic reticulum, Golgi apparatus, polysomes, and mitochondria. Asexual development involves aerial hyphae growing upward from the colony, followed by production of conidiophores and mature conidia from the tips of aerial hyphae. Asexual development structures were first seen at 15 hr and mature conidia at 27 hr (Kasuga and Glass 2008).

We mapped expression for 20 GPCRs, present in four groups (Figure 4A). Group 1 genes are highly expressed in leading basal hyphae and tend to decrease in expression in older parts of the colony (Figure 4A). The only exceptions are *gpr-2* and *gpr-37*, which exhibit elevated expression at 27 hr. Δ*gpr-2* mutants have defects in aerial hyphae development (Table 1), consistent with increased expression of *gpr-2* later during colony development. Group 2 (four genes) has a pattern of high expression early and late during colony development (Figure 4A). This group includes two genes (*gpr-3* and *gpr-30*) that are required for normal aerial hyphae development. The expression of Group 3 genes peaked late during asexual sporulation (27 hr) at the time when conidia are mature. Of these four genes, mutants lacking *gpr-23* have defects in aerial hyphae development (Table 1; also see below). Lastly, Group 4 genes have low levels of expression early during colony development, with most increasing from 3−21 hr and then falling again at 27 hr (Figure 4A). This group includes two genes (*gpr-16* and *gpr-39*) that are required for normal aerial hyphae development (Table 1).

Expression data for a time course of asexual development was mined from microarray data (Greenwald *et al.* 2010) (Figure 4B). RNA was extracted from the entire colony at 0, 2, 4, 8, 10, and 24 hr. At 12 hr, the basal hyphae (bottom) were collected separately from the upper portion of the colony containing aerial hyphae and conidia. This was done to detect expression of genes in the cell types that give rise to aerial hyphae and mature conidia. Only the top of the colony was analyzed at 14 and 18 hr, to focus on genes expressed in aerial hyphae and conidia. We were able to detect expression of four GPCR genes (*gpr-8*, *gpr-23*, *gpr-17*, and *nop-1*) in this dataset (Figure 4B).

Mutants lacking *gpr-23* have a defect in both hyphal growth and asexual development (Table 1). Therefore, it is of interest that *gpr-23* expression is highest during the early stages of colony establishment from 0 to 4 hr, the time during which aerial hyphae production is initiated. As supported by previous work (Bieszke *et al.* 2007), *nop-1* is a late-stage conidiation gene with high transcript levels from 12 to 24 hr in aerial hyphae tissue (Greenwald *et al.* 2010). Δ*nop-1* mutants have defects in aerial hyphae development (Table 1).

We next analyzed RNAseq expression data for strains grown in two different carbon sources: sucrose and crystalline cellulose (Avicel) (Figure 5) (Coradetti *et al.* 2012). Sucrose is the carbon source used for VM medium and, with the exception of testing growth on Avicel (see below), was present in all media used for phenotypic analysis in our study. We detected expression for 40 GPCR genes in the dataset (Figure 5). Since we had tested available mutants for increased or decreased growth relative to wild type on Avicel (Table 2), we were able to compare growth phenotypes and gene expression for numerous GPCR genes.

The majority of GPCR genes (24) were expressed to greater levels on Avicel relative to sucrose (Figure 5). Among these, 10 genes (*nop-1*, *gpr-3*, *gpr-8*, *gpr-11*, *gpr-18*, *gpr-24*, *gpr-30*, *gpr-31*, *gpr-34*, and *gpr-39*) had mean expression levels greater than 200 reads per kilobase per million mapped reads on Avicel (Figure 5). From this highly expressed group, *nop-1* had the greatest fold difference of 166, whereas *gpr-34* was second, with a 32-fold difference comparing Avicel *vs.* sucrose. Of interest, 60% of the genes with the greatest expression levels on Avicel are annotated as Pth11-related. As described previously, our analysis revealed that three GPCR mutants (Δ*gpr-32*, Δ*gpr-36*, and Δ*gpr-39*; all Pth11-related class) had a phenotype during growth on Avicel (Table 2). mRNA levels for all three of these genes are greater on Avicel than sucrose (Figure 5), with *gpr-39* levels increased sixfold on Avicel *vs.* sucrose). These observations suggest that the other GPCRs that are highly expressed on Avicel may be required for growth on this carbon source, but that gene redundancy is masking phenotypes in single mutants. This is of particular interest for the Pth11-related group, as 14 out of the 22 detected genes are expressed to higher levels on Avicel than sucrose (Figure 5) and this class of GPCRs has the most members that are required for normal growth on Avicel.

There are nine genes (*gpr-9*, *gpr-10*, *orp-1*, *gpr-17*, *gpr-22*, *gpr-23*, *gpr-29*, *gpr-35*, and *gpr-37*) for which the mRNA amount is at least twofold greater during growth on sucrose *vs.* Avicel (Figure 5). This set includes the two mPR-like GPCRs in *N. crassa* (*gpr-9* and *gpr-10*; Table 1). In particular, when cultured on sucrose, *gpr-10* is the most highly expressed GPCR in the dataset. Δ*gpr-10* (and Δ*gpr-9*) mutants had no growth or developmental phenotypes on VM medium, perhaps reflecting gene redundancy between these two mPR-like GPCRs in *N. crassa*.

### Phylogenetic analysis of *N. crassa* Pth11-related GPCRs

**Figure 6:**
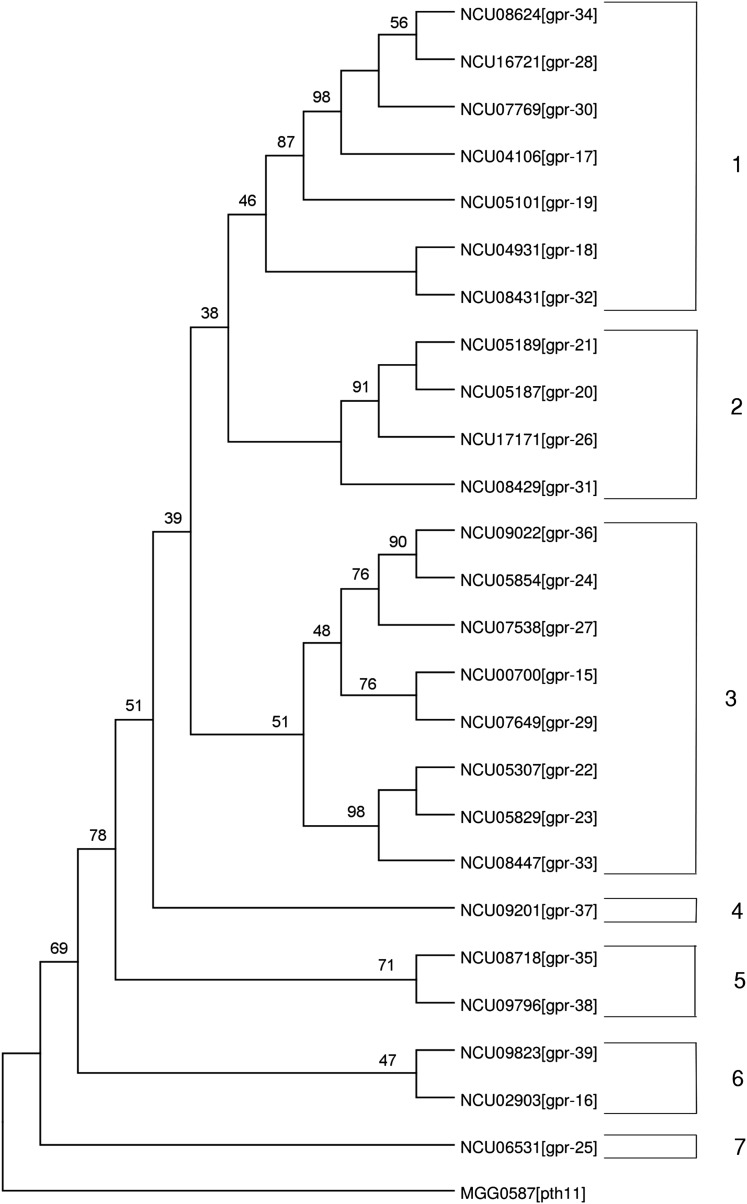
Phylogenetic analysis of Pth11-related proteins in *N. crassa*. The protein sequences for the 25 *N. crassa* Pth11-related proteins from Li *et al.* (2007) (Table 1) and *M*. oryzae Pth11 (outgroup) were aligned using T-coffee and trimmed using TrimAl. The consensus parsimony tree was produced using 100 bootstrap replicates. Branch lengths are indicated on the left side of the figure, whereas groups of related proteins (1−7) are indicated on the right. See the section *Materials and Methods* for details.

The 25 *N. crassa* Pth11-related GPCR protein sequences were aligned with the *M. oryzae* Pth11 protein sequence (MG05871) (see the section *Materials and Methods*). The alignment was adjusted manually to account for conservation of the CFEM domains present in *N. crassa* GPR-25 and *M. oryzae* Pth11. The CFEM domain consists of a conserved sequence containing eight cysteines that is proposed to play an important role in fungal pathogenesis (Kulkarni *et al.* 2003). GPR-25 is the only *N. crassa* GPCR with a CFEM domain and is the protein most similar to *M. oryzae* Pth11 in *N. crassa* (Figure 6).

A phylogenetic tree was generated from the alignment data (Figure 6). A parsimony tree was built in the original *M. oryzae-N. crassa* analysis (Kulkarni *et al.* 2005), so a similar approach was taken with the *N. crassa* sequences in this study. The tree revealed seven distinct groups among the *N. crassa* Pth11-related proteins. In Group 1, three Pth11-related genes were highly expressed on Avicel (expression greater than 200 FPKM; *gpr-18*, *gpr-30*, and *gpr-34*; Figure 5). A mutant was not available for *gpr-18*, and the Δ*gpr-30* was normal, but the Δ*gpr-32* strain had a phenotype when grown on Avicel. Therefore, not only does Group 1 have protein-based homology, but its members may function in processing Avicel.

The tree revealed eight clusters, each containing two closely related proteins. Inspection of the characteristics of several of these pairs revealed shared patterns of gene expression and/or phenotypes that suggest overlapping functions. For example, *gpr-18* and *gpr-32* cluster together in Group 1. Both genes are more highly expressed on Avicel relative to sucrose (Figure 5); are similarly expressed during sexual development (Group 5; Figure 3); and are in neighboring expression clusters for colony development (Groups 3 and 4; Figure 4A). Although the mutant for *gpr-18* was not available, Δ*gpr-32* mutants have a phenotype on Avicel (Table 2).

*gpr-36* and *gpr-24* are related proteins in Group 3. Both genes are more highly expressed on Avicel than sucrose (Figure 5) but have differing patterns of expression during sexual development (Figure 3). Δ*gpr-36* has a phenotype on Avicel, whereas Δ*gpr-24* has a chemical phenotype on fludioxonil (Table 1). *gpr-15* and *gpr-29* branch together in Group 3. These two genes have similar patterns of expression during sexual development (Figure 3) but exhibit opposite trends during growth on sucrose *vs.* Avicel (Figure 5). Importantly, although Δ*gpr-15* and Δ*gpr-29* mutants both have reduced linear growth rates, Δ*gpr-15* uniquely possesses defects in sexual development and Δ*gpr-29* in aerial hyphae extension (Table 1). These results may indicate that these two genes have overlapping functions not only during linear hyphal growth, but also possibly asexual and sexual development.

*gpr-22* and *gpr-23* are another closely related pair in Group 3. Both genes are more highly expressed on sucrose than Avicel (Figure 5), are in the same expression group during sexual development (Group 2; Figure 3), and share phenotypic defects in aerial hyphae development (Table 1). Expression of *gpr-23*, but not *gpr-22*, was detected during the time course of asexual development in two microarray experiments (Figure 4, A and B). These results strongly suggest a role for these two genes in asexual development and Avicel utilization.

*gpr-16* and *gpr-39* comprise Group 6. These genes are coexpressed during the time courses for sexual development and colony growth (Figure 3; Figure 4A). Both mutants have defects in aerial hyphae development, coinciding with their expression later during colony development (Table 1; Figure 4A). Of interest, these two proteins are the most closely related to GPR-25, the Pth11 homolog in *N. crassa*. As mentioned previously, Δ*gpr-25* mutants are resistant to the actin depolymerizing agent cytochalasin A. The observation of functions for *gpr-16* and *gpr-39* in aerial hyphae development may indicate similarities in regulation of this process and the infectious appressorium in *M. oryzae* by G protein signaling. Furthermore, the phenotype of Δ*gpr-25* mutants may indicate a role for actin polymerization in this process.

Our phylogenetic results have some similarities but also differ from those of Kulkarni *et al.* 2005, who performed analysis using all *M. oryzae* and *N. crassa* Pth11-related proteins. As mentioned previously, GPR-25 is the closest *N. crassa* homolog to *M. oryzae* Pth11 in both studies (Kulkarni *et al.* 2005) (Figure 6). In addition, GPR-16 (NCU02903) and GPR-39 (NCU09823) are the nearest *N. crassa* relatives to *N. crassa* GPR-25 (Figure 6). However, there are several instances of closely related pairs of *N. crassa* proteins that did not cluster together in the Kulkarni study (*e.g.*, GPR-18 and GPR-32; GPR-24 and GPR-36), possibly due to the absence of the *M. oryzae* proteins in our analysis or the different method used to produce the tree.

## Discussion

We have analyzed available mutants for annotated GPCR genes in *N. crassa*. Of the 36 available mutants, 29 (81%) exhibited at least one phenotypic defect, and 15 of these are members of the Pth11-related class. In addition, 59% (10/17) of the GPCR genes with a phenotype in one the three major growth/developmental pathways analyzed were Pth11-like. Specifically, 17% (1/6) of sexual development, 57% (8/14) of aerial hyphae and 100% (5/5) of hyphal growth mutants lacked Pth11-related genes, revealing the functional importance of this group, particularly for hyphal growth and aerial hyphae development.

A recent study of GPCR knockout mutants in the filamentous fungal pathogen *Aspergillus flavus* analyzed 15 mutants in Groups I-IX (Affeldt *et al.* 2014). *N. crassa* has 14 genes in these groups. The *A. flavus* study did not include Groups X-XIII or the Pth11-related class (named class XIV in our study), corresponding to 31 genes in *N. crassa* and the majority of predicted GPCRs in filamentous fungi. Although *N. crassa* mutants for groups VII and XIII were not available for our study, we are the first to systematically analyze mutants in the Pth11-related class of predicted GPCR genes in a filamentous fungus. Only a small subset of the phenotypic analyses addressed in the *A. flavus* study overlapped with those in our experiments, including hyphal extension on sucrose-containing medium and sensitivity to hydrogen peroxide and sodium chloride (Affeldt *et al.* 2014). Inspection of the data showed there was no overlap in the three phenotypes for the corresponding *A. flavus* and *N. crassa* mutants. It is difficult to draw strong conclusions from such a small set of phenotypes, and further experimentation is necessary to investigate evolutionarily conserved functions for predicted GPCRs in the two species.

Our results with GPCR genes contrast with those obtained for analysis of other *N. crassa* gene groups implicated in early steps in signal transduction in eukaryotes: serine-threonine protein kinases and serine-threonine and tyrosine protein phosphatases (Park *et al.* 2011b; Ghosh *et al.* 2014). The number of predicted GPCRs (43) is intermediate between serine-threonine protein kinases (77) and protein phosphatases (30). The fraction of GPCR mutants with a growth rate phenotype (14%) is much lower than that observed for kinase (42%) and phosphatase (50%) mutants. This result suggests either that GPCRs are not critical for growth rate regulation or the presence of gene redundancy. Likewise, no single GPCR mutant was absolutely required for female fertility in *N. crassa* in both mating types, whereas complete female sterility was observed for 39 of the kinase and four of the phosphatase mutants. In contrast to the results for growth rate and sexual cycle phenotypes, the proportion of predicted GPCR mutants with defects in asexual development (39%) is similar to that observed for protein kinase mutants (40%), but less than that for protein phosphatase (58%) mutants. In terms of the fraction of mutants with at least one growth, developmental or chemical/nutritional phenotype, GPCRs are intermediate (81%) between the phosphatases (100%) and kinases (71%). Closer inspection of phenotype classes indicates that in comparison with GPCRs, a larger proportion of kinase and phosphatase mutants are multiply defective in the three major growth/developmental pathways. This result may reflect greater gene redundancy in the GPCRs and/or their involvement in functions that were not assayed during our investigations.

We used existing RNA profiling data from three datasets to mine expression trends for GPCR genes during perithecial development, colony growth, conidiation and growth on Avicel. We were able to analyze 37 of 43 genes (86%) from the sexual development time course, 20 of 43 (47%) in the colony establishment dataset, 4 of 43 (9%) during the conidiation time course, and 40 of 43 genes during expression on Avicel *vs.* sucrose (93%). We observed several instances in which the expression and phenotypic data appeared to overlap. From the colony development time course data, we noted that the CRL GPCR *gpr-2* is expressed in the older parts of the colony (Figure 4A). Consistent with this observation, Δ*gpr-2* mutants have defects in aerial hyphae development (Table 1). During the conidiation time course, we saw that mRNA levels for the Pth11-like GPCR gene *gpr-23* are greatest during the time aerial hyphae production is initiated (Figure 4B), and mutants lacking this gene have defects in basal hyphae extension and aerial hyphae development (Table 1).

We also noted instances in which only one gene in a larger group of coexpressed genes yielded a phenotype for that process. For example, during the sexual development time course, although the majority of Group 5 genes are highly expressed at the time of perithecial maturation (Figure 3), only two genes in this group, the CRL *gpr-2* and the pheromone receptor *pre-1*, have sexual development defects (Table 1). The finding that the other 10 genes have no obvious sexual cycle phenotype suggests that at least some of them may be functionally redundant.

As previously mentioned, 20 genes were available for expression analysis on Avicel and sucrose. Of interest, 14 of the 20 available (70%) were Pth11-class genes that were expressed to higher levels on Avicel than sucrose. This observation, along with the fact that all three of the mutants (Δ*gpr-32*, Δ*gpr-36*, and Δ*gpr-39*) with defects during growth on Avicel were Pth11-related genes, suggests that this group of predicted GPCRs may function in sensing or utilization of plant-derived biomass. In the phylogenetic tree (Figure 6) Group 1 was particularly noteworthy, as three of the genes (*gpr-34*, *gpr-30*, and *gpr-18)* were highly expressed on Avicel and one member (not highly expressed; *gpr-32*) had a phenotype on Avicel. We hypothesize that construction and analysis of strains carrying multiple knockout mutations in Group 1 genes may reveal additional genes with phenotypes on Avicel.

The identification of GPCR mutants with resistance to cytochalasin A may be related to the requirement for actin during endocytic recycling of GPCRs [reviewed in (Engqvist-Goldstein and Drubin 2003)]. Presumably, loss of a GPCR that is actively endocytosed during hyphal growth in wild type would result in greater tolerance to an actin destabilizing agent, such as cytochalasin A. Alternatively, these GPCRs may be involved in signaling pathways that direct the relative amount or positioning of actin cables or patches during hyphal growth (Berepiki *et al.* 2011).

In this study we examined the expression and functions of predicted GPCR genes during growth and development in *N. crassa*. Although none of the four transcriptomic data sets included data for every predicted GPCR, all contained data for the gene in at least one unavailable mutant. We obtained expression data for many GPCR genes that did not yield a phenotype when mutated. In several cases, closely related genes shared expression patterns, but only one mutant had a phenotype, suggesting overlapping functions for the two genes. These findings can be addressed in the future through construction of strains carrying multiple GPCR gene knockouts. Special interest was taken in the Pth11-related class due to its influence on asexual development and hyphal growth. This study has expanded knowledge of a gene family related to a pathogenicity locus in a fungal plant pathogen using an easily tractable non-pathogenic fungal species.

## Supplementary Material

Supporting Information
