# Supplementary material for: Global Analysis of Predicted G Protein−Coupled Receptor Genes in the Filamentous Fungus, Neurospora crassa
Source: G3 (Bethesda). 2015 Oct 9;5(12):2729–43. doi: 10.1534/g3.115.020974 (PMC4683645; doi:10.1534/g3.115.020974)
Supplement: Supporting Information [file supp_5_12_2729__index.html]

Global Analysis of Predicted G Protein−Coupled Receptor Genes in the Filamentous Fungus, Neurospora crassa — Supporting Information 

# Global Analysis of Predicted G Protein−Coupled Receptor Genes in the Filamentous Fungus, *Neurospora crassa*

## Supporting Information for Cabrera *et al.*, 2015

**Files in this Data Supplement:**

- Table S1 - Comprehensive data for growth and developmental phenotypes (.xlsx, 16 KB).
